# Ethyl Butyrate Synthesis Catalyzed by Lipases A and B from *Candida antarctica* Immobilized onto Magnetic Nanoparticles. Improvement of Biocatalysts’ Performance under Ultrasonic Irradiation

**DOI:** 10.3390/ijms20225807

**Published:** 2019-11-19

**Authors:** Rodolpho R. C. Monteiro, Davino M. Andrade Neto, Pierre B. A. Fechine, Ada A. S. Lopes, Luciana R. B. Gonçalves, José C. S. dos Santos, Maria C. M. de Souza, Roberto Fernandez-Lafuente

**Affiliations:** 1 Departamento de Engenharia Química, Universidade Federal do Ceará, Campus do Pici, Bloco 709, CEP 60455760, Fortaleza 60000-000, CE, Brazil; montteirorrc@gmail.com (R.R.C.M.); lrg@ufc.br (L.R.B.G.); 2 Departamento de Química Analítica e Físico-Química, Universidade Federal do Ceará, Campus do Pici, Bloco 940, CEP 60455760, Fortaleza 60000-000, CE, Brazil; davinomachado@gmail.com (D.M.A.N.); fechine@ufc.br (P.B.A.F.); 3 Instituto de Engenharias e Desenvolvimento Sustentável, Universidade da Integração Internacional da Lusofonia Afro-Brasileira, Campus das Auroras, CEP 62790970, Redenção 68550-000, CE, Brazil; ada@unilab.edu.br; 4 Departamento de Biocatálisis, ICP-CSIC, Campus UAM-CSIC Cantoblanco, 28049 Madrid, Spain

**Keywords:** ethyl butyrate, lipase A from *Candida antarctica*, lipase B from *Candida antarctica*, magnetic nanoparticles, ultrasonic irradiation

## Abstract

The synthesis of ethyl butyrate catalyzed by lipases A (CALA) or B (CALB) from *Candida antarctica* immobilized onto magnetic nanoparticles (MNP), CALA-MNP and CALB-MNP, respectively, is hereby reported. MNPs were prepared by co-precipitation, functionalized with 3-aminopropyltriethoxysilane, activated with glutaraldehyde, and then used as support to immobilize either CALA or CALB (immobilization yield: 100 ± 1.2% and 57.6 ± 3.8%; biocatalysts activities: 198.3 ± 2.7 U*_p_*_-NPB_/g and 52.9 ± 1.7 U*_p_*_-NPB_/g for CALA-MNP and CALB-MNP, respectively). X-ray diffraction and Raman spectroscopy analysis indicated the production of a magnetic nanomaterial with a diameter of 13.0 nm, whereas Fourier-transform infrared spectroscopy indicated functionalization, activation and enzyme immobilization. To determine the optimum conditions for the synthesis, a four-variable Central Composite Design (CCD) (biocatalyst content, molar ratio, temperature and time) was performed. Under optimized conditions (1:1, 45 °C and 6 h), it was possible to achieve 99.2 ± 0.3% of conversion for CALA-MNP (10 mg) and 97.5 ± 0.8% for CALB-MNP (12.5 mg), which retained approximately 80% of their activity after 10 consecutive cycles of esterification. Under ultrasonic irradiation, similar conversions were achieved but at 4 h of incubation, demonstrating the efficiency of ultrasound technology in the enzymatic synthesis of esters.

## 1. Introduction

The catalysis of reactions mediated by biological catalysts is an alternative to traditional chemical catalysis [1,2,3]. Enzymes are biological macromolecules that reduce the occurrence of undesirable side reactions and can work under mild temperature and pressure, which minimizes the energy demand and thermal degradation of the product of interest [4,5].

In this sense, lipases (triacylglycerol hydrolases, EC 3.1.1.3) are enzymes that stand out due to the wide range of reactions that they can catalyze (e.g., hydrolysis, acidolysis, esterification, transesterification, and amination) in different media (organic solvents, ionic liquids, supercritical fluids, etc.) [6,7,8], with high stability and great diversity [9,10].

Lipase A (CALA) and lipase B (CALB) can be isolated from the yeast *Candida antarctica* [11,12]. CALB is the most widely used lipase, being employed in the cosmetic, pharmaceutical, food, and beverage industries [13,14,15,16,17,18]. On the other hand, CALA has not hitherto been so widely used, even though it bears several interesting features [19].

CALA (45 kDa, isoelectric point 7.5 and optimum pH 7.0) is an extremely thermostable protein (optimum temperature above 90 °C), whereas CALB has a lower stability (optimum temperature around 40 °C) [19,20]. CALA displays activity using large substrates (bulky alcohols, esters and tertiary alcohols) [21,22], and it is possible to accommodate sterically hindered substrates [23]. CALB (33 kDa, isoelectric point 6.0 and optimum pH 7.0) has a very small lid [24]. Both enzymes possess the classical serine hydrolases catalytic triad [20,25].

However, the industrial application of lipases is limited due to problems concerning their stability, recovery and reuse. A solution to these problems is their immobilization on insoluble solid supports [26]. A proper immobilization strategy, together with simplifying the enzyme reuse or use in continuous reactor, may improve enzyme stability, activity, selectivity or specificity; purify the enzyme; or decrease its inhibition [27,28,29,30,31,32].

In the case of CALB, there is an immobilized commercial preparation supplied by Novozymes, Novozym^®^ 435, immobilized on a macroporous acrylic polymer resin (Lewatit VP OC 1600) [33]. This biocatalyst has been successfully used in diverse reactions, but it has been described to possess some limitations, like enzyme release in certain media, polymer dissolution in organic media, or the capture of hydrophilic compounds in the enzyme environment [33,34,35,36,37,38,39]. Thus, an alternative support may further increase the implementation of these interesting enzymes. For example, lipase immobilization on magnetic nanoparticles (MNPs) allows easy recovery of the enzyme by application of a magnetic field [2]; besides, the enzyme will be immobilized on the external area of the nanoparticle and this avoids any kind of diffusional limitation. This also raised some problems (e.g., the enzyme is exposed to all inactivating external interfaces [28]). Moreover, these supports have a high surface area, may be easily modified to introduce groups with chemical reactivity, and have a high thermal stability [40,41,42]. Therefore, magnetic nanoparticles have become versatile and interesting immobilization supports to achieve enzyme immobilization/stabilization via multipoint covalent attachment; aminated supports can be easily activated using glutaraldehyde [43].

Covalent attachment is a very convenient method for enzymatic immobilization [44,45,46,47,48]. Glutaraldehyde is a very widely used method to immobilize enzymes on primary amino functionalized supports [49,50,51]. The supports activated with glutaraldehyde are very versatile, due to the presence of hydrophobic, ionic and chemically reactive moieties [43,50,51]. In amino glutaraldehyde preactivated supports, the enzyme–support interactions are very strong and the leaching of the enzyme is minimized, which produces a more stable biocatalyst [47].

Short chain fatty acids are used as aroma and flavors and may be extracted from natural products, but at a very high price and low yield [52]. The industrial production of these esters employs acidic catalysts. This implies a great demand for energy and the promotion of side reactions, causing environmental problems and requires complex downstream development [53,54,55]. Moreover, the final products cannot be labeled as natural [56]. In contrast, enzymatic catalysis allows the production of products that can be labelled as natural and the reaction is performed under mild conditions with lower environmental impact [57]. Among these esters, ethyl butyrate is applied as an aroma and fragrance compound in beverage, food, cosmetic, and pharmaceutical industries [2,51,58].

The direct enzymatic esterification between an acid and an alcohol is called thermodynamically controlled synthesis [59]. The yields are independent from the catalysts, which only determine the feasibility of the process (e.g., the thermodynamic yields may not be reached if the catalyst is inactivated or inhibited). The main problem of this reaction is water production. If water is captured (e.g., using molecular sieves), the yields may be shifted towards the synthesis, and also this may permit preventing the formation of an aqueous phase on the biocatalyst beads (where the water is formed). This water layer can inhibit or inactivate the enzyme [60]. This has been solved using very hydrophobic supports and also using ultrasonic irradiation to stir inside the biocatalyst and to prevent this aqueous phase formation [61,62,63,64].

Ultrasound is the use of sonic energy at frequencies >20 kHz and requires a medium to propagate [65]. The consecutive cycles of compression and rarefaction of sound waves can cause a phenomenon known as cavitation, which comprises the formation, enlargement and collapse of bubbles, which, when it occurs near the limit phase of two immiscible liquids, can provide a very efficient stirring, facilitating mass transfer; furthermore, the collapse of the bubbles causes supercritical conditions of temperature and pressure [66,67].

On the other hand, optimization of the process when some variables can co-interact is not simple using a standard optimization protocol due to the high number of experiments that this may require [68]. In this sense, the statistical methodologies of design, such as the Central Composite Design (CCD), allow the study of the covariance of the variables (independent factors) to obtain the best response (dependent factor) with a reduced number of runs [69].

Therefore, the main objective of this communication is to synthesize ethyl butyrate using lipases A or B from *Candida antarctica* immobilized onto iron magnetic nanoparticles coated with 3-aminopropyltriethoxysilane and activated with glutaraldehyde, (CALA-MNP and CALA-MNP). Fixing an acid concentration of 1.0 M, the influence of temperature, reaction time, molar ratio, and content of the biocatalyst were evaluated in the production of this flavor ester by a CCD. The thermodynamic parameters of the reactions (variations of enthalpy and entropy and Gibbs free energy) were also established as well as the operational stability of the enzymatic biocatalyst.

## 2. Results and Discussion

### 2.1. Immobilization Performance

As it is shown in Table 1, for the covalent immobilization (1 mg of protein for 1 g of support, pH 7.0, 25 mM and 25 °C), the immobilization yield was 100% for CALA-MNP and 57.6% for CALB-MNP; for both biocatalysts, a reference solution was prepared (an enzyme solution under identical conditions to those of immobilization but in absence of support) and it maintained full activity during all immobilization processes, so the immobilization yield could be calculated by the decrease of activity in the supernatant. The performance of immobilization was evaluated by recovery activity as well, presenting a value of 97.5% for CALA-MNP and 80.5% for CALB-MNP. As it is seen in Table 1, for the adsorption immobilization, both biocatalysts performed worse than the glutaraldehyde activated nanoparticles, showing that glutaraldehyde plays a significant role in enzyme immobilization.

The support used in this communication, having a high surface density of amino groups and activated with glutaraldehyde, may permit hydrophobic, anionic exchange and covalent interactions with the enzyme [30,70]. Lipases may become immobilized onto the support first via a rapid interfacial activation, which is faster than the direct covalent attachment; however, the use of ionic strength and detergents may ensure immobilization firstly via direct covalent attachment [51].

At neutral pHs, glutaraldehyde groups are more stable than at alkaline pHs and the most reactive group in the enzyme tends to be the terminal amino group [71]. Moreover, if more enzyme nucleophile groups are exposed to the support, other covalent bonds between the enzyme and support may be established [72], enabling multipoint covalent immobilization.

### 2.2. Characterization of Fe_3_O_4_ NPs

**Figure 1:**
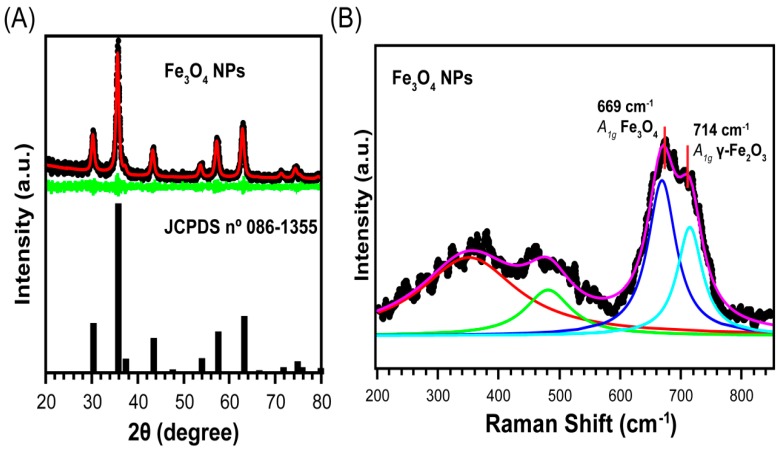
(**A**) XRD patterns of the Fe_3_O_4_ NPs synthesized herein; black dots and the red and green lines represent the measurement data, adjusted diffractogram and subtraction between the measurement data and adjusted diffractogram, respectively. (**B**) Raman spectrum of the Fe_3_O_4_ NPs; black dots and the pink line are the measurement data and the Lorentzian fit, respectively. Other colored lines are adjusted vibrational modes of the Fe_3_O_4_ NPs. Further details are given in Section 3.

XRD and Raman spectroscopy analysis were performed to confirm the success in the synthesis of the magnetic core of the biocatalysts, as shown in Figure 1. It was noticed by XRD analysis that the magnetic core is composed by a single structural phase, cubic inverse spinel structure *Fd3m*, which is characteristic of Fe_3_O_4_ and γ-Fe_2_O_3_ (see Figure 1A). The cubic cell lattice parameter for our sample was 8.371 (5) Å, which is an intermediate value between Fe_3_O_4_ and γ-Fe_2_O_3_, indicating a partially oxidized Fe_3_O_4_ NPs (8.396 Å for Fe_3_O_4_ and 8.346 Å for γ-Fe_2_O_3_) [73,74]. The crystallite size of the sample was calculated by Scherrer’s equation. A diameter of 13.0 (2) nm was found, indicating the preparation of a nanoparticulate material.

Even though XRD was not conclusive in relation to the different phases of the iron oxides, Raman spectroscopy was able to differentiate Fe_3_O_4_ from γ-Fe_2_O_3_ [75]. Figure 1B shows a typical Raman spectrum for nanoparticles with broad peaks and relatively low signal-to-noise ratio. The band centered in 669 cm^−1^ can be attributed to *A*_1g_ phonon mode of Fe_3_O_4_, while a shoulder in 714 cm^−1^ corresponds to the *A*_1g_ mode of γ-Fe_2_O_3_. Additionally, the modes in 350 and 432 cm^−1^ can be assigned to *T*_2g_ and *E*_g_ of γ-Fe_2_O_3_ [76]. The presence of these aforementioned vibrational modes confirms that the magnetic core of the biocatalysts prepared herein is composed by Fe_3_O_4_ and γ-Fe_2_O_3_.

### 2.3. Fourier-Transform Infrared Spectroscopy (FT-IR)

**Figure 2:**
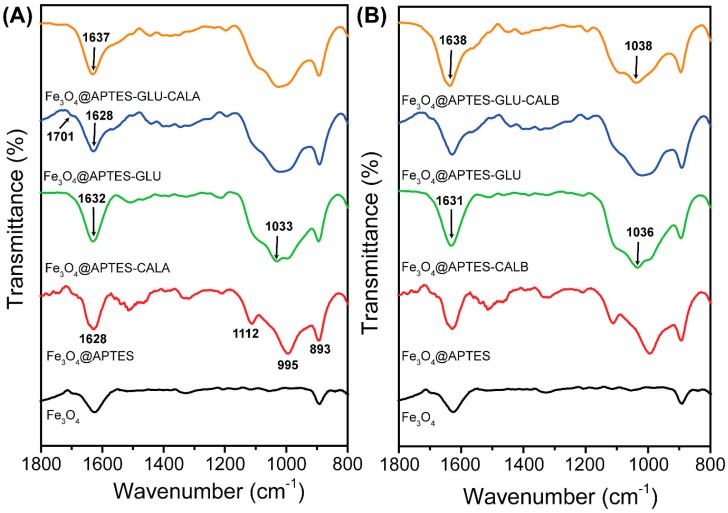
FT-IR spectra for the nanoparticles obtained in this communication. In each column is shown the series of spectra for (**A**) CALA and (**B**) CALB immobilization.

Fourier-transform Infrared Spectroscopy (FT-IR) analysis of all samples was performed aiming to confirm the success of the modifications proposed in this communication. In Figure 2, the FT-IR spectra in the range of 800–1800 cm^−1^ are shown. All samples presented a set of bands in 490–770 cm^−1^ relative to the Fe–O stretching of the Fe_3_O_4_ MNPs, which corroborates with results of DRX and Raman spectroscopy (see Figure 1). After functionalization with APTES, a set of bands in 893, 995 and 1112 cm^−1^ were evidenced, which are attributed to Si–O–H and Si–O–Si groups of APTES [77] ((Figure 2A,B), spectrum of Fe_3_O_4_@APTES). Furthermore, amine groups of APTES onto the surface of Fe_3_O_4_ NPs can be evidenced by the vibrational mode in 1628 cm^−1^, relative to NH_2_ bending [77]. Non-covalent immobilization of CALA and CALB on the surface of Fe_3_O_4_@APTES can be confirmed by the appearance of the vibrational modes in 1632–1631 and 1033–1036 cm^−1^ relative to amide I and stretching of C–N, respectively ((Figure 2A,B), spectrum of Fe_3_O_4_@APTES-CALA and Fe_3_O_4_@APTES-CALB) [78]. The activation of the sample Fe_3_O_4_@APTES with GLU is confirmed by the presence of the bands in 1628, 1701 and 2862–2930 cm^−1^, which are attributed to the stretching of C = N, C = O and C–H groups, respectively [79,80]. Moreover, the presence of CALB and CALA, after the covalent immobilization onto Fe_3_O_4_@APTES-GLU, is confirmed by the bands of amide I and II in 1637–1638 and 1537–1544 cm^−1^, respectively [78]. Additionally, for the spectra of the samples Fe_3_O_4_@APTES-GLU-CALA and Fe_3_O_4_@APTES-GLU-CALB, it was evidenced by a diminishing/disappearance of the C = O stretching in 1701 cm^−1^ due to the reaction between the aldehyde groups of Fe_3_O_4_@APTES-GLU and residual amine groups of CALB and CALA. Therefore, the results of FT-IR corroborate the success of the modifications of the surface of Fe_3_O_4_ MNPs and preparation of the biocatalysts.

### 2.4. Model Fitting and ANOVA

In order to determine the optimal enzyme content, molar ratio, temperature, reaction time, and substrate molar ratio for the synthesis of ethyl butyrate using heptane and solvent and CALA-MNP or CALB-MNP as biocatalysts, a CCD was carried out and the results are presented in Table 2. For both biocatalysts, the highest conversion (96.3 ± 2.1% for CALA-MNP and 92.3 ± 2.7% for CALB-MNP) was obtained for run 19 (10 mg of biocatalyst, 1:1, 45 °C and 6 h).

At a confidence interval of 95%, the statistical analysis of the model was done by Fisher’s statistical test for analysis of variance. For both biocatalysts, the computed F-values (91.76 for CALA-MNP and 26.22 for CALB-MNP) were highly significant (*p* < 0.0001). The model fitting was checked by the determination coefficient (R^2^) and correlation coefficient (R). The determination coefficient (R^2^ = 0.99 for CALA-MNP and R^2^ = 0.97 for CALB-MNP) implies that sample variation of up to 95% for both biocatalysts is attributed to the independent variables and can be explained by the model. For this study, both biocatalysts presented a value of R over 0.90 (*r* = 0.97 for CALA-MNP and *r* = 0.93 for CALB-MNP), suggesting a satisfactory representation of the process and an excellent correlation between the experimental results and the theoretical values predicted by the model polynomial equation, which is given below.

(1) $$\begin{array}{cc} Y_{CALA-MNP}= & -145.33+6.95X_{1}-0.35X_{1}^{2}+3.61X_{2}-1.06X_{2}^{2}+3.93X_{3}+0.04X_{3}^{2}\\ & \;+37.1X_{4}-2.78X_{4}^{2}-0.02X_{1}X_{2}+0.02X_{1}X_{3}+0.03X_{1}X_{4}-0.01X_{2}X_{3}\\ & \;-0.67X_{2}X_{4}-0.04X_{3}X_{4} \end{array}$$

(2) $$\begin{array}{cc} \;Y_{CALB-MNP}= & -119.56+6.43X_{1}-0.30X_{1}^{2}-8.34X_{2}+0.37X_{2}^{2}+4.87X_{3}-0.05X_{3}^{2}\\ & \;+38.22X_{4}-2.08X_{4}^{2}-0.01X_{1}X_{2}+0.01X_{1}X_{3}-0.04X_{1}X_{4}+0.01X_{2}X_{3}\\ & \;-0.37X_{2}X_{4}-0.02X_{3}X_{4}\; \end{array}$$

In which, Y is the conversion for the esterification reaction; and X_1_, X_2_, X_3_, and X_4_ are the coded values of enzyme content, molar ratio, temperature, and reaction time, respectively.

For the synthesis of ethyl butyrate biocatalyzed by CALA-MNP, the optimal conditions were found to be 10 mg of biocatalyst, 1:1 (butyric acid/ethyl alcohol), obtaining a theoretical value of 100% for the conversion predicted by the model after 6 h of reaction at 45 °C and 150 rpm. For CALB-MNP, the optimal conditions were found to be 12.5 mg of the biocatalyst, 1:1 (butyric acid/ethyl alcohol), obtaining a theoretical yield of 98.3% after 6 h of incubation at 45 °C and 150× *rpm*. Under the optimized conditions, experimental validation of the proposed model was conducted. After three repetitions of the procedure by titration, the conversion obtained was 99.2 ± 0.3% (CALA-MNP) and 97.5 ± 0.8% (CALB-MNP), which shows an excellent correlation between the experimental and statistically predicted results.

In a similar study, Souza et al. (2017) evaluated the esterification of butyric acid with ethyl ethanol by CALB-MPN for the synthesis of ethyl butyrate, achieving conversions over 90% after 8 h of incubation at 25 °C and 150× *rpm* for 10 mg of CALB-MNP and 1:1 (acid/alcohol) [2]. Other authors evaluated the synthesis of ethyl butyrate biocatalyzed by lipase from *Rhizomucor miehei* immobilized onto chitosan; as a result, a conversion over 90% was achieved after 6 h of incubation at 25 °C and 150× *rpm* for 200 mg of biocatalyst and 1:1 (acid/alcohol) [81]. Therefore, the optimized operational conditions obtained in this communication for the synthesis of ethyl butyrate are in accordance with those reported in the literature; however, slightly higher conversions were achieved for both biocatalysts under study at higher temperatures.

**Figure 3:**
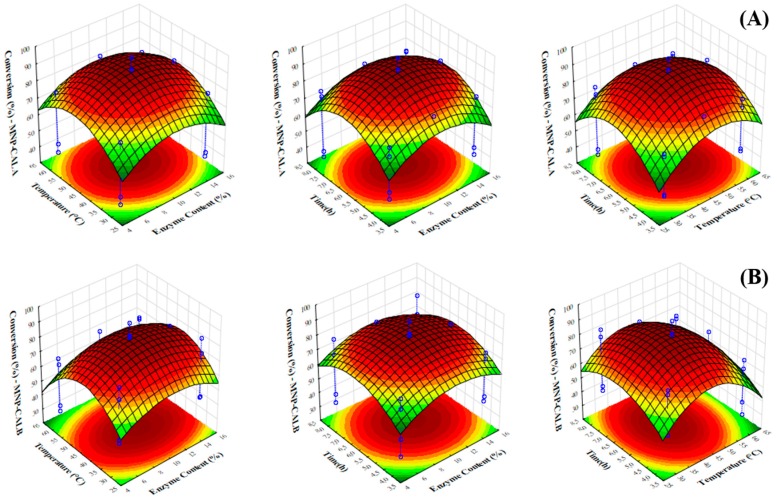
Fitted response surfaces. (**A**) CALA-MNP; (**B**) CALB-MNP. Reaction medium: CALA-MNP or CALB-MNP (5–15 mg), butyric acid (1.0 mol/L), ethyl alcohol 1:1–1:5 (butyric acid/ethyl alcohol), and heptane (reaction volume = 1.0 mL). The reactions were performed for 4–8 h at 30–60 °C and 150× *rpm*. Further details are given in Section 3.

In order to evaluate the influence of biocatalyst content, molar ratio (butyric acid/ethyl alcohol), temperature and time on the synthesis of ethyl butyrate biocatalyzed by CALA-MNP or CALB-MNP, surface response methodology were plotted, as can be seen in Figure 3.

For CALA-MNP, temperature was the only independent variable that positively affected the conversion, meaning that changing the level of the variable from −1 to 1 (or from 30 to 60 °C) significantly increased the response. According to Figure 3A, it can be stated that conversion increases with temperature for the studied temperature range until a certain level. Indeed, temperature is a crucial factor for enzymatic biocatalysis, since it increases the solubility of reactants in the reaction medium, may reduce medium viscosity, and in that way, reduces mass transfer limitations; besides, it facilitates with the molecular collision interface, decreasing the energy barrier between the molecules of reactants and enabling the formation of an enzyme–substrate complex, which causes an increase in the initial rate of reaction and, therefore, causes an improvement in the conversion [82,83]. Enzymes are stable at a certain temperature range, overpassing this, the increase in temperature leads to thermal denaturation of the enzyme molecule [84].

For CALB-MNP, biocatalyst content was the independent variable that most positively affected the conversion. According to Figure 3B, it can be stated that conversion tends to increase with increasing enzyme content. Indeed, increasing the biocatalyst content may positively affect rates of reactions catalyzed by immobilized enzymes, provided that they do not aggregate forming large pellets with high diffusion limitations [83,85].

Molar ratio was the independent variable that most affected the conversion for both biocatalysts (CALA-MNP and CALB-MNP). In fact, under optimized conditions, increasing the amount of ethyl alcohol for both systems decreased the conversion by a factor of 1.3 for 1:4 (acid/alcohol). A large amount of ethanol may decrease the conversion due to its polar character, which shows hydrophilic interaction with the water layer present on the surface of the enzyme and may cause changes in the macromolecule structure with consequent inhibition and reduction of enzyme activity [86]. During the first step of esterification, ethyl alcohol at higher concentrations may promote its binding to the enzyme, decreasing the concentration of butyric acid in the vicinity of the enzyme and, therefore, reducing the reaction rate [87].

### 2.5. Time Course of Esterification

**Figure 4:**
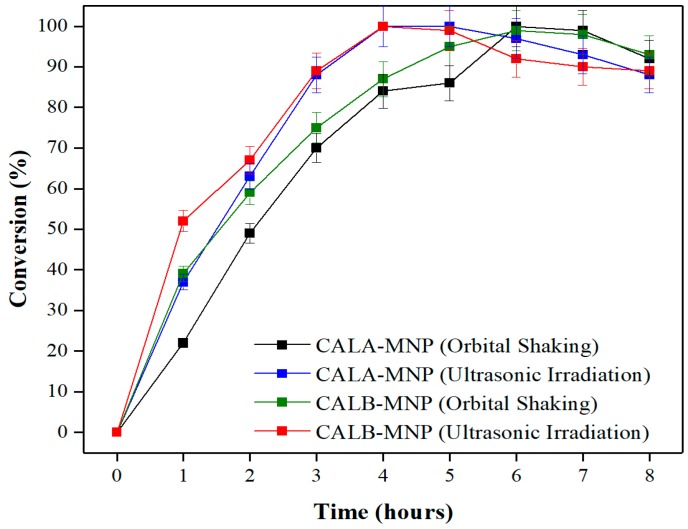
Esterification course for ethyl butyrate synthesis biocatalyzed by CALA-MNP or CALB-MNP under orbital shaking (150× *rpm*) or ultrasonic irradiation (37 kHz and 300 W). Reaction medium: CALA-MNP (10 mg) or CALB-MNP (12.5 mg), 1:1 (butyric acid/ethyl alcohol) and heptane (reaction volume = 1.0 mL). The reactions were performed for 1–8 h at 45 °C. Further details are given in Section 3.

Once the optimal conditions for the production of ethyl butyrate catalyzed by CALA-MNP or CALB-MNP were determined by the experimental design and then validated, the conversion for the time varying from 1 to 8 h for orbital shaking and ultrasonic irradiation techniques was analyzed, as is shown in Figure 4.

As can be seen in Figure 4, for CALA-MNP, the highest conversion achieved for the orbital shaking technique was 100 ± 2.9% after 6 h of incubation. For the ultrasonic irradiation technique, the same conversion (100 ± 1.6%) was achieved after 4 h. A similar behavior was observed for CALB-MNP. Indeed, for the orbital shaking technique, the highest conversion (99 ± 3.3%) was achieved after 6 h of incubation, whereas for the ultrasonic irradiation technique, the highest conversion (100 ± 1.2%) was achieved after 4 h.

Using an immobilized lipase, Badgujar and Bhanage (2015) synthetized benzyl butyrate, anisyl butyrate and o-cresyl butyrate by using the ultrasonic irradiation technique (33 kHz and 100 W) and obtained a conversion of 99% after 3 h of incubation at 52 °C. In the same conditions, the authors synthetized these flavor esters without using ultrasonic irradiation and obtained conversions of 57%, 55% and 43% for benzyl butyrate, anisyl butyrate and o-cresyl butyrate, respectively [86].

Therefore, ultrasonic irradiation may favor the synthesis of flavor esters catalyzed by immobilized lipases by achieving higher conversions at smaller reaction times, when compared to the traditional mechanical techniques. In fact, the ultrasonic irradiation creates supercritical conditions of temperature and pressure, besides a very efficient agitation, which facilitates mass transfer and allows the dissociation of water molecules and dissolved oxygen, creating highly reactive free radicals (-OH and -OOH) [85,88]. Nevertheless, it is known that ultrasonic cavitation may cause changes in protein conformation, affecting its catalytic activity [89]. Indeed, high frequencies and potencies can cause denaturation of enzymes, while low frequencies and potencies will not result in the desired effect [90].

For this communication, the value of frequency (37 kHz) and power (300 W) favored the enzymatic synthesis of ethyl butyrate biocatalyzed for immobilized enzymes (CALA-MNP or CALB-MNP).

### 2.6. Thermodynamics of the Enzymatic Esterification

By employing the van’t Hoff equation (Equation (5)), the variations in enthalpy (ΔH) and entropy (ΔS) were determined from the angular and linear coefficient, respectively, of the straight line of ln $K_{\mathrm{EQ}}$ versus reciprocal temperature. The graphic method utilized in this communication, as it is known, is such a rough estimation to determine such thermodynamics properties; however, a more rigorous method involves quantum mechanics, which is very difficult to apply to biological systems and is not the purpose of this communication [91]. Table 3 shows the variation in enthalpy (ΔH) and entropy (ΔS) for the enzymatic synthesis of ethyl butyrate biocatalyzed by CALA-MNP or CALB-MNP under orbital shaking or ultrasonic irradiation. The regression coefficients (R^2^) for all values were over 0.90.

As can be seen in Table 3, for all systems, the positive values of enthalpy variation (ΔH) imply the endothermic nature of the esterification system; therefore, an increase in temperature is expected to favor product formation [92]. Indeed, for this communication, increasing the temperature has increased the $K_{\mathrm{EQ}}$ value, since higher temperatures intensify successful collisions of the substrates with the active site of the enzymes [93]. Similarly, for all systems, the positive values of the entropy change (ΔS) imply that the esterification system is becoming increasingly disordered as the temperature increases; besides, the formation of water molecules, as a byproduct of esterification, increases the disorder of the system [93].

For both biocatalysts under study, the variations in enthalpy and entropy were approximately twice bigger for the ultrasonic irradiation system, when compared to the orbital shaking one. The variation in enthalpy depends on the structure of the enzyme and its capacity of formation and disruption of chemical bonds [94], whereas the variation in entropy is associated with the number of molecules of the substrates with sufficient energy to react [95,96]. In fact, at 37 kHz, 300 W and for the range of temperature under study (30 to 45 °C), the ultrasonic irradiation has favored the synthesis of ethyl butyrate biocatalyzed by immobilized lipases. Yet, for temperatures over 45 °C, the conversion decreased increasing temperature. Ultrasound irradiation at low frequencies is not able to inactivate enzymes [97], but the combined effects of ultrasound irradiation (frequency and power) and temperature (over 45 °C) may have led to the progressive inactivation of the proteins, since the conversion decreased increasing temperature.

**Figure 5:**
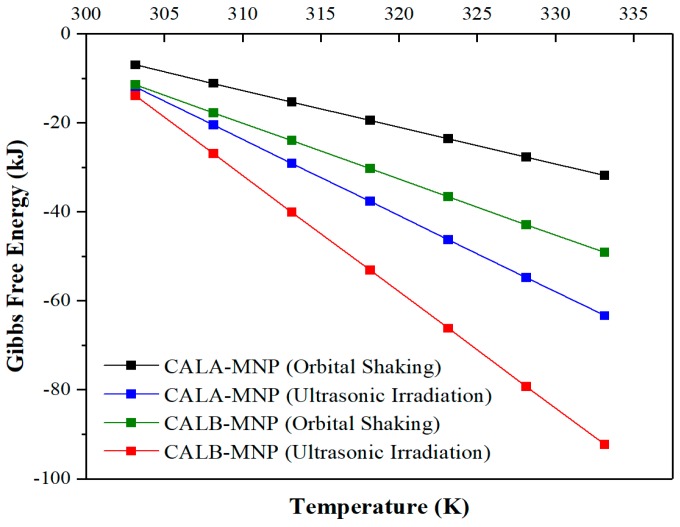
Gibbs free energy change for the enzymatic esterification as a function of temperature biocatalyzed by CALA-MNP or CALB-MNP under optimal conditions at orbital shaking (150× *rpm*) and ultrasonic irradiation (37 kHz, 300 W). Further details are given in Section 3.

Once the variation in enthalpy and entropy were calculated, it was possible to determine the Gibbs free energy by Equation (4), considering an interval of temperature from 303 to 333 K. Figure 5 shows the Gibbs free energy for the range of temperature under study [98].

### 2.7. Operational Stability

**Figure 6:**
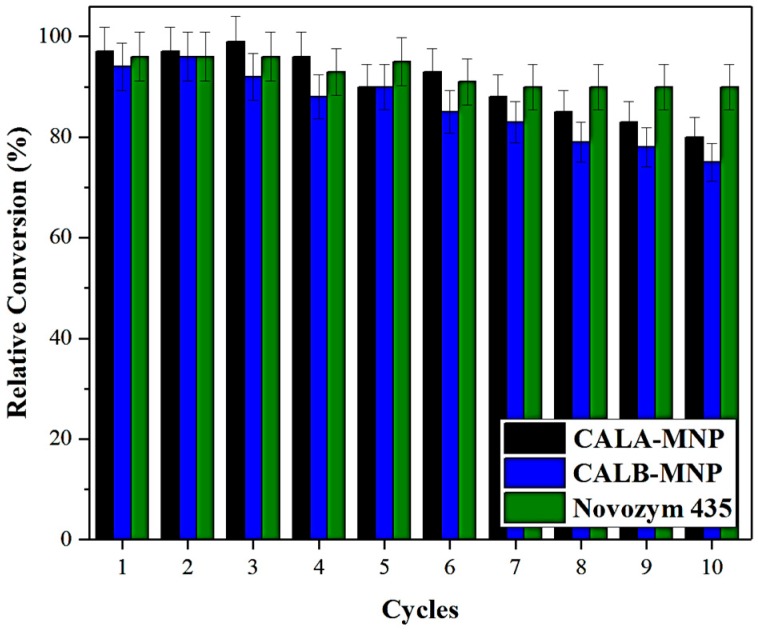
Operational Stability. Black column: CALA-MNP; blue column: CALB-MN; green column: Novozym® 435. Reaction medium: CALA-MNP: 10 mg of biocatalyst, 1:1, 6 h of incubation at 45 °C and 150× *rpm*; CALB-MNP and Novozym^®^ 435: 12.5 mg of biocatalyst, 1:1, 6 h of incubation at 45 °C and 150× *rpm*. Further details are given in Section 3.

The operational stability of CALA-MNP and CALB-MNP were evaluated for the synthesis of ethyl butyrate under optimized conditions. Novozym^®^ 435 was used for comparison purposes. To do so, consecutives cycles of esterification were performed, as is shown in Figure 6.

Enzymes have higher prices than chemical catalysts; thus, to be competitive, immobilized enzymes should be able to be reused several times, maintaining operational stability [99]. In fact, even though enzymes are very sensitive to changes in the environment, the immobilization of such biological macromolecules may promote their rigidification, which keeps the enzyme active for longer periods of time, allowing consecutive reuses [99]. As is shown in Figure 6, none of the biocatalysts have a very significant drop in activity after 10 consecutive cycles of esterification.

**Figure 7:**
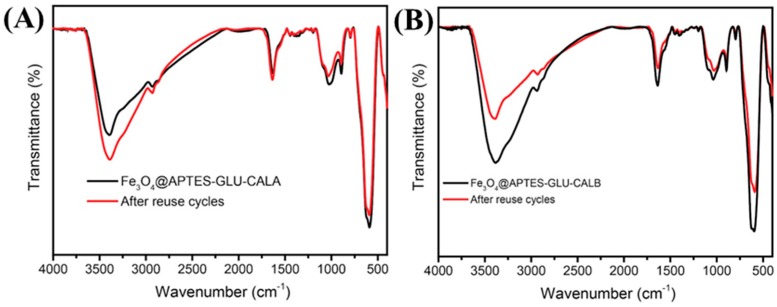
FT-IR spectra for CALA-MNP (**A**) and CALA-MNP (**B**) before and after 10 consecutive reuses. Further details are given in Section 3.

In order to evaluate if any chemical change happened after the reuse of the biocatalysts, FT-IR analysis of the samples Fe_3_O_4_@APTES-GLU-CALA and Fe_3_O_4_@APTES- GLU-CALB after the last cycle was performed. As is shown in Figure 7, structural changes in the spectra of the biocatalysts after reuse is not evident. All bands relative to the Fe_3_O_4_ core and modifications that were made remained in the spectra after reuse. Therefore, the covalent immobilization prevented any leaching of CALA or CALB from Fe_3_O_4_@APTES-GLU and any decrease in activity over the consecutive cycles of esterification may be related to the deactivation of the enzymes by temperature, solvents, substrate, and mechanical agitation.

## 3. Materials and Methods

### 3.1. Materials

Lipases A (20.88 mg/mL) and B (7.88 mg/mL) from *Candida antartica* were kindly donated by Novozymes (Alcobendas, Spain). Lipase B from *Candida antartica* immobilized on acrylic resin (Novozym^®^ 435), 3-aminopropyltriethoxysilane (APTES), glutaraldehyde solution grade II 25% (*w*/*v*), and *p*-nitrophenyl butyrate (*p*-NPB) were purchased from Sigma-Aldrich (Sigma-Aldrich, St Louis, MI, USA). Iron magnetic nanoparticles (Fe_3_O_4_) were produced by the co-precipitation method. The chemical reagents used for this synthesis were FeCl_3_.6H_2_O (pure granulated 99%), FeSO_4_.7H_2_O (pure granulated 99%) and 30% ammonia solution, supplied by Sigma-Aldrich (Sigma-Aldrich, St Louis, MI, USA). All reagents of analytical grade were purchased from Synth (São Paulo, Brazil) and Vetec (São Paulo, Brazil).

### 3.2. Methods

#### 3.2.1. Ultrasound Equipment Setup

The equipment used was an ultrasonic bath (Unique Inc., model USC 2800A, São Paulo, Brazil). The equipment presents a capacity volume of 9.5 L with the following dimensions: 300 × 240 × 150 mm (length × width × height). Two disc transducers were placed at the bottom of the reactor. The ultrasonic frequency was 37 kHz and the total ultrasonic power 300 W. Additionally, the equipment has temperature control.

#### 3.2.2. Synthesis of Iron Magnetic Nanoparticles (Fe_3_O_4_) Functionalized with 3 Aminopropyltriethoxysilane (APTES)

Firstly, 2.5 g of FeSO_4_·7H_2_O (9 mmol) and 4.0 g of FeCl_3_·6H_2_O (15 mmol) were dissolved in 30 mL of deionized water under mechanical stirring at 60 °C for 30 min. Then, 40 mL of concentrated ammonium hydroxide were added to the iron cations solution. The system remained at 60 °C under mechanical stirring for more than 30 min. Afterwards, the black nanoparticles were washed several times with deionized water and three times with ethanol.

For the functionalization with APTES, non-functionalized Fe_3_O_4_ NPs were dispersed in 300 mL of ethanol (95%), under room temperature, using an ultrasound bath (37 kHz and 300 W) for 1 h. Subsequently, 10 mL of APTES were added to the nanoparticle’s dispersion. The system continued with sonication for more than 1 h. Finally, functionalized nanoparticles were washed four times with ethanol and dried under vacuum.

#### 3.2.3. Activation of Fe_3_O_4_@APTES with Glutaraldehyde (GLU)

The amino terminal groups of Fe_3_O_4_@APTES were activated with glutaraldehyde (Fe_3_O_4_@APTES-GLU) to promote covalent attachment between the enzyme and the support, via formation of an imine bond. To do so, 250 μL of glutaraldehyde were placed in direct contact with 0.1 g of previously dried Fe_3_O_4_@APTES. The mixture was kept under constant stirring for 1 h at 25 °C and, after that, it was washed three times with sodium phosphate buffer solution (25 mM and pH 7.0) to remove the excess of glutaraldehyde [100].

#### 3.2.4. Covalent Immobilization of CALA or CALB onto Fe_3_O_4_@APTES-GLU

Lipase A from *Candida antartica* (CALA, 20.88 mg/mL) and lipase B from *Candida antartica* (CALB, 7.88 mg/mL) were immobilized on Fe_3_O_4_@APTES-GLU, here named Fe_3_O_4_@APTES-GLU-CALA and Fe_3_O_4_@APTES-GLU-CALB, respectively, (CALA-MNP and CALB-MNP, respectively, for short) by covalent attachment. To do so, 0.1 mg of Fe_3_O_4_@APTES-GLU were suspended in 10 mL of sodium phosphate buffer solution (25 mM and pH 7.0) containing CALA or CALB (enzyme loading: 1 mg/g of support) in the presence of 0.01% Triton X-100. The system was kept under constant stirring for 2 h at 25 °C. Finally, the immobilized lipases were separated from the solution by magnetic decantation and washed with sodium phosphate buffer solution (25 mM and pH 7.0) to neutrality. The amount of enzyme immobilized on the support was determined by measuring the initial and final concentration of CALA or CALB in the supernatant of the immobilization suspension [44].

#### 3.2.5. Adsorption Immobilization of CALA or CALB onto Fe_3_O_4_@APTES

Lipase A from *Candida antartica* (CALA, 20.88 mg/mL) and lipase B from *Candida antartica* (CALB, 7.88 mg/mL) were immobilized on Fe_3_O_4_@APTES-GLU, here named Fe_3_O_4_@APTES-GLU-CAL and Fe_3_O_4_@APTES-GLU-CALB, respectively, (CALA-MNP and CALB-MNP, respectively, for short) by adsorption. To do so, 0.1 g of Fe_3_O_4_@CHI-GLU were suspended in 10 mL of sodium phosphate buffer solution (25 mM and pH 7.0) containing CALA or CALB (enzyme loading: 1 mg/g of support) in the presence of 0.01% Triton X-100. The immobilization process was similar to that described in the previous section.

#### 3.2.6. Determination of Enzymatic Activity and Protein Concentration

The activity of the soluble and immobilized enzyme was determined by the hydrolysis of *p*-NPB as a substrate; the *p*-nitrophenol concentration was quantified spectrophotometrically at 348 nm. Activity measurements were performed in sodium phosphate buffer solution (25 mM and pH 7.0) at 25 °C, from the measurement of *p*-nitrophenol released during the hydrolysis of 0.5 mM *p*-NPB (ε = 10.052 mol/cm under these conditions) [101]. To initiate the reaction, 50 μL of suspended lipase solution was added to 50 μL of *p*-NPB and 2.5 mL of the buffer solution. An international unit of activity (U) was defined as the amount of enzyme that hydrolyzes 1 μmol of *p*-NPB per minute under the conditions described above. The protein concentration was measured by the Bradford method [102], and bovine serum albumin was used as reference.

#### 3.2.7. Immobilization Parameters

The performance of the immobilized enzyme was evaluated by the immobilization parameters described by Silva et al. [103]. In short, the immobilization yield (IY) was defined as the ratio between the activity of enzymes retained on the support (initial activity–final activity) and initial activity. The theoretical activity (At_T_) was calculated using the immobilization yield (IY) and the enzyme load. The recovery activity (At_R_) was determined as the ratio between the biocatalyst activity (At_B_) and theoretical activity (At_T_).

#### 3.2.8. X-Ray Diffraction (XRD)

X-Ray Diffraction (XRD) profiles were collected by a X’Pert MPD X-ray powder diffractometer (PANalytical, Westborough, MA, USA) with 40 kV and 30 mA in a scanning range of 2θ = 20–80° equipped with a Co Kα tube. The diffraction patterns were obtained using Bragg-Brentano geometry in continuous mode with a speed of 0.5°/min and step size of 0.02° (2θ). The Rietveld structure refinement was used to interpret and analyze the diffraction data using the program DBWstools 2.4 [104]. The full width at half maximum (FWHM) of the instrument was calculated with the standard lanthanum hexaboride. Parameters extracted from the refinement, both the percentage of errors (15.25%) and goodness of fitting values (0.86), were found to be in agreement with those of a high-quality refinement [105]. The crystallite size of each sample was calculated using Scherrer’s equation.

#### 3.2.9. Raman Spectroscopy

Raman spectra was recorded using a LabRAM HR (HORIBA Scientific, Kyoto, Japan). The spectral excitation was performed with a laser using a 632.8 nm line, with an adjustable D1 filter with effective powers of 10.7 μW with three accumulations of 10 s. Previous to the adjustment, the spectrum was mathematically adjusted by a Savitzky–Golay filter.

#### 3.2.10. Fourier-transform Infrared Spectroscopy (FT-IR)

The functionalized MNPs and enzyme immobilization were studied by Fourier-transform infrared spectroscopy (FT-IR). The spectra were recorded by using a Shimadzu model 8300 spectrophotometer. The samples were ground in an agate mortar and pressed into pellets of KBr. The range used was 400–4000 cm^−1^, with a resolution of 2 cm^−1^ and 128 scans.

#### 3.2.11. Enzymatic Esterification

The synthesis of ethyl butyrate by esterification was conducted in a reaction medium containing heptane, butyric acid (1.0 mol/L) and ethanol for molar varying from 1:1 to 1:5 (acid/alcohol), totalizing a reactional medium of 1 mL. A biocatalyst mass (CALA-MNP or CALB-MNP) of 5–15 mg was added to initiate the reaction, which was carried out under orbital shaking (150× *rpm*), at 30–60 °C for time varying from 4 to 8 h. Conversion was monitored by determining the acidity index through the Ca 5–40 AOCS method [104].

#### 3.2.12. Central Composite Design (CCD)

In order to determine the optimal conditions for the synthesis of ethyl butyrate, a central composite design (CCD) of four variables (enzyme content, molar ratio, temperature, and reaction time) was carried out. Table 4 shows the four variables, each at three levels. The design was performed by 24 tests at factorial points and three with repetitions at the central point.

The adjusted experimental data is shown by the proposed model in Equation (1), which considers the linear, quadratic and interaction effects among the variables, and it was used to plot response surfaces for all variables.

(3) $$Y={\;\beta }_{0}+\;\sum \beta_{i}X_{i}+\;\sum \beta_{i}X_{i}X_{j}+\;\sum X_{\mathrm{ii}}X_{i\;}^{2}$$

In which, *Y* is the response variable; *β_0_* is the constant; *β_i_, β_ii_, and β_ij_* are the coefficients for the linear, quadratic, and interaction effects, respectively; and *X_i_* and *X_j_* are the coded levels of variables *X_i_* and *X_j_*.

#### 3.2.13. Statistical Analysis

The softwares Statistica^®^ 10 (Statsoft, Tulsa, OK, USA) and OriginPro 2017 (OriginLab, Northampton, MA, USA) were used for the experimental design and statistical analysis for the ethyl butyrate production process. The statistical analysis of the model was performed by analysis of variance (ANOVA). The significance of the regression coefficients and the associated probabilities, p(t), were determined by Student’s t-test; the second order model equation significance was determined by Fisher’s F-test. The variance explained by the model is given by the determination coefficients, R^2^.

#### 3.2.14. Operational Stability

Operational stability was evaluated by consecutive reactions for the synthesis of ethyl butyrate under optimized conditions. Prior to each cycle, the biocatalysts were separated from the reaction medium by magnetization and washed three times with hexane to remove unreacted products and substrates.

#### 3.2.15. Thermodynamic Properties

For the purpose of this study, it was considered that there was enough time for the reaction to achieve equilibrium [91]. The apparent equilibrium constant, *K_EQ_*, may be obtained by Equation (4) for equimolar stoichiometry.

(4) $$K_{\mathrm{EQ}}=\frac{\left[W\right]\left[E\right]}{\left[\mathrm{AC}\right]\left[\mathrm{AL}\right]}=\frac{x^{2}}{{\left(1-x\right)}^{2}}$$

In which, [W], [E], [AC], and [AL] represent, respectively, the water, ester, acid, and alcohol concentration; and *x* represents the equilibrium conversion. The equilibrium constant dependence of temperature is given by the equation of van’t Hoff (Equation (5)).

(5) $$\ln K_{\mathrm{EQ}}=\frac{-\Delta H}{\mathrm{RT}}+\frac{\Delta S}{R}$$

In which, ΔH (J/mol), ΔS (J/mol.K), R (J/mol.K), and T (K) represent enthalpy variation, entropy variation, universal gas constant, and temperature, respectively. Once the enthalpy and entropy variation for the reaction were obtained, the Gibbs free energy was estimated by Equation (6). In which, ΔG represents the free energy of Gibbs (J/mol).

(6) ΔG=ΔH−TΔS

## 4. Conclusions

The synthesis of ethyl butyrate biocatalyzed by lipases A or B from *Candida antarctica* immobilized onto magnetic nanoparticles (functionalized with APTES and activated with glutaraldehyde) is a promising alternative for the production of such a flavor ester, when compared to the traditional industrial processes of extraction and production using chemical catalysts, since CALA-NPM and CALB-MNP exhibit high specificity and selectivity and have the possibility of easy reuse of these biocatalysts. Besides, by the ultrasound-assisted synthesis, it was possible to achieve conversions (over 97% for both biocatalysts) similar to those achieved by orbital shaking, but at a smaller reaction time (6 h against 4 h), demonstrating the efficiency of ultrasonic irradiation for the synthesis of ethyl butyrate biocatalyzed by immobilized lipases.

## Figures and Tables

**Table 1:** Immobilization parameters of CALA and CALB: immobilization yield (IY), theoretical biocatalyst activity (At_T_), biocatalyst activity (At_D_) and recovery activity (At_R_) (1 mg of protein per 1 g of support was the loading of the supports, and the experiments were performed in 0.25 mM sodium phosphate at pH 7 and 25 °C). Further details are given in Section 3.

Biocatalyst	IY (%)	At_T_ (U/g)	At_D_ (U/g)	At_R_ (%)
Fe_3_O_4_@APTES–GLU–CALA	100 ± 1.2	203.3 ± 1.2	198.3 ± 2.7	97.5 ± 1.9
Fe_3_O_4_@APTES-CALA	80 ± 3.1	110.7 ± 3.1	46.2 ± 3.3	41.7 ± 3.2
Fe_3_O_4_@APTES–GLU–CALB	57.6 ± 3.8	65.7 ± 3.8	52.9 ± 1.7	80.5 ± 3.2
Fe_3_O_4_@APTES-CALB	38.2 ± 3.3	43.6 ± 3.3	31.3 ± 1.2	71.3 ± 2.2

**Table 2:** Coded values and results for the CCD. Further details are given in Section 3.

Run	X_1_	X_2_	X_3_	X_4_	Conversion (%)	
					CALA-MNP	CALB-MNP
1	−1	−1	−1	−1	65.4 ± 1.4	70.5 ± 2.6
2	−1	−1	−1	1	72.3 ± 1.0	78.3 ± 2.0
3	−1	−1	1	−1	70.5 ± 0.3	63.3 ± 1.8
4	−1	−1	1	1	75.4 ± 0.4	67.9 ± 0.1
5	−1	1	−1	−1	40.0 ± 1.2	42.8 ± 0.2
6	−1	1	−1	1	35.5 ± 1.0	40.7 ± 2.0
7	−1	1	1	−1	44.2 ± 0.7	31.1 ± 3.3
8	−1	1	1	1	38.8 ± 0.2	34.7 ± 0.0
9	1	−1	−1	−1	63.8 ± 0.4	73.5 ± 1.0
10	1	−1	−1	1	76.9 ± 1.0	83.3 ± 2.7
11	1	−1	1	−1	75.8 ± 1.9	69.1 ± 1.7
12	1	−1	1	1	77.1 ± 1.7	70.0 ± 2.9
13	1	1	−1	−1	40.8 ± 0.3	43.1 ± 0.9
14	1	1	−1	1	38.6 ± 0.2	43.8 ± 1.1
15	1	1	1	−1	45.2 ± 1.4	39.8 ± 1.6
16	1	1	1	1	40.2 ± 0.2	36.6 ± 2.8
17	−1	0	0	0	77.3 ± 2.0	71.2 ± 2.5
18	1	0	0	0	84.0 ± 1.6	77.7 ± 1.4
19	0	−1	0	0	96.3 ± 2.1	92.3 ± 2.7
20	0	1	0	0	73.8 ± 2.4	74.7 ± 2.9
21	0	0	−1	0	75.4 ± 2.0	65.3 ± 2.4
22	0	0	1	0	85.6 ± 2.0	73.4 ± 2.9
23	0	0	0	−1	76.1 ± 1.2	70.0 ± 2.0
24	0	0	0	1	80.2 ± 1.5	77.4 ± 2.2
25(C)	0	0	0	0	88.1 ± 1.0	83.2 ± 1.0
26(C)	0	0	0	0	89.1 ± 0.0	83.8 ± 1.4
27(C)	0	0	0	0	89.6 ± 0.1	83.3 ± 0.1

**Table 3:** Variation of enthalpy and entropy for the synthesis of ethyl butyrate biocatalyzed by CALA-MNP or CALB-MNP under optimal conditions of orbital shaking (150× *rpm*) and ultrasonic irradiation (37 kHz, 300 W). Further details are given in Section 3.

	CALA-MNP		CALB-MNP	
	Orbital Shaking	Ultrasonic Irradiation	Orbital Shaking	Ultrasonic Irradiation
ΔH (kJ/mol)	223.1	507.8	369.9	779.1
ΔS (kJ/mol·K)	0.8	1.7	1.3	2.6

**Table 4:** Variables and their levels for the CCD.

Variable	Name	Coded Levels		
		−1	0	+1
X_1_	Enzyme Content (mg)	5	10	15
X_2_	Molar Ratio (acid/alcohol)	1:1	1:3	1:5
X_3_	Temperature (°C)	30	45	60
X_4_	Time (h)	4	6	8
